# Prenatal testosterone does not explain sex differences in spatial ability

**DOI:** 10.1038/s41598-018-31704-y

**Published:** 2018-09-12

**Authors:** Teemu Toivainen, Giulia Pannini, Kostas A. Papageorgiou, Margherita Malanchini, Kaili Rimfeld, Nicholas Shakeshaft, Yulia Kovas

**Affiliations:** 1 Department of Psychology, Goldsmiths, University of London, London, United Kingdom; 2 Social, Genetic and Developmental Psychiatry Centre, King’s College, London, United Kingdom; 3 School of Psychology, Queen’s University Belfast, Belfast, United Kingdom; 4 Department of Psychology, Tomsk State University, Tomsk, Russia; 5 Department of Psychology, University of Texas at Austin, Austin, TX, USA

## Abstract

The most consistent sex differences in cognition are found for spatial ability, in which males, on average, outperform females. Utilizing a twin design, two studies have shown that females with male co-twins perform better than females with female co-twins on a mental rotation task. According to the Twin Testosterone Transfer hypothesis (TTT) this advantage is due to in-uterine transmission of testosterone from males to females. The present study tested the TTT across 14 different spatial ability measures, including mental rotation tasks, in a large sample of 19–21-year-old twins. Males performed significantly better than females on all spatial tasks, with effect sizes ranging from *η*^2^ = 0.02 to *η*^2^ = 0.16. Females with a male co-twin outperformed females with a female co-twin in two of the tasks. The effect sizes for both differences were negligible (*η*^2^ < 0.02). Contrary to the previous studies, our results gave no indication that prenatally transferred testosterone, from a male to a female twin, influences sex differences in spatial ability.

## Introduction

Sex differences are small to negligible in most cognitive traits^1,2^. However, some measures show differences between males and females^3–6^. The most consistent finding of cognitive sex differences comes from spatial ability, in which men, on average, consistently outperform women^7,8^. This finding has been replicated in large cross-cultural samples^9,10^. As spatial skills have shown positive correlations with academic and career success in the Science, Technology, Engineering and Mathematics (STEM) disciplines^11^, they may partly account for the current underrepresentation of women in these areas^12,13^.

Spatial ability can be described as the ability to produce, recall, store, and modify spatial relations among objects^14^ and to visualize the transformation of these relations due to changes, for example, in perspective^15–17^. Spatial ability is a component of general cognitive ability, alongside many others, such as working memory, verbal ability and processing speed^18^. However, spatial ability also involves aspects differentiable from general cognitive ability^18,19^. Conceptually, spatial ability is often described as having several, separate components, although their definitions overlap^19^. This may be partly due to the complex nature of the tasks, requiring many parallel cognitive processes, such as apprehending and encoding spatial forms^20^. However, two recent studies, using data from a large longitudinal twin sample in the United Kingdom (also used in this study), have shown evidence for a uni-factorial structure of spatial ability across a variety of different spatial measures, both phenotypically and genetically^19,21^. In both studies, the first order factor explained approximately 42 per cent of the variance across diverse spatial tasks.

Previous studies have shown that males outperform females in several spatial ability measures^7^. For example, studies on spatial navigation have showed large sex differences favoring men^22,23^. Also, on a mental rotation task (MRT), males outperform females by almost one standard deviation^7^. A MRT is an example of a widely-used measure of spatial visualization, which involves complex, multi-stage manipulations of spatial information^24^. This difference has been found consistently across several cultures^6^ and it has been documented in infants as young as 3–5 months^25^. However, one spatial task in which females perform better than males is object location memory. A meta-analysis found an overall effect size of *d* = 0.27 favoring females over males in a memory-dependent spatial task of remembering object locations^6^.

The factors that contribute to sex differences in spatial ability are still poorly understood. Both biological and environmental factors have been proposed to initiate and maintain the sex differences in spatial ability. Environmental explanations have highlighted the role of previous experiences and learning environments^26,27^, whereas biological investigations have concentrated on genetic and hormonal effects^28–31^. To date, behavioral genetic studies have only found small^32^, or non-existent^33^, sex differences in etiology of any cognitive abilities. In line with this, studies on the etiology of spatial ability found only negligible differences in genetic and environmental factors driving sex differences in spatial ability in males and females^19^, including in mental rotation^28^.

Hormonal effects, which are influenced by genes and environments, are a biological mechanism affecting spatial cognition^34,35^. The sex hormone testosterone, necessary for sexual development and sexual behavior, is also present in brain areas associated with cognitive abilities such as language and spatial ability^36,37^. Some evolutionary arguments suggest that testosterone is a factor maintaining spatial sex differences^34^. According to such accounts, the greater elaboration of the neurocognitive basis of spatial ability, especially in 3-dimensional environments, is due to navigating and tracking movement that had more evolutionary relevance for males than females^34,35^. In line with the evolutionary argument, naturally occurring testosterone levels vary between sexes: typical testosterone levels in clinical assessment, measured in blood, range between 0.5 to 2.4 nmol/L in females, and from 10 to 38 nmol/Lin males^38^.

Several studies have investigated the effect of testosterone on individual differences in spatial ability within sexes. Studies have shown that better spatial ability was associated with elevated testosterone levels, both due to natural fluctuations and extraneous administration^39–42^. For example, a study showed that females with higher levels of testosterone performed significantly better than females with lower testosterone levels on a visual maze task^39^. Other studies have reported the relationship between extraneous administration of testosterone and improvement in spatial ability task performance. The effect has been reported among older men^40^; female-to-male transsexuals^41^; and young women^42^. However, not all studies have supported the association between elevated testosterone levels and better spatial ability performance among females. One study found no within-sex associations between mental rotation task and endogenous, non-fluctuating testosterone when measured in blood^43^. Among males, the studies on the relationship between the level of testosterone and spatial ability performance have shown mixed results. Some studies have reported that high levels of testosterone are negatively associated with spatial ability among males^44,45^. One study tested and supported a curvilinear relationship between testosterone levels and spatial ability performance among young adults, suggesting that after exceeding an optimal level, additional testosterone may impair spatial performance^46^.

Prenatal testosterone may also influence cognitive development: it affects brain functions and neural structure during early prenatal development^47,48^. One line of evidence for the association between elevated prenatal testosterone levels and increased spatial ability in females comes from clinical samples. Congenital Adrenal Hyperplasia (CAH) is a genetic condition that elevates fetal testosterone levels. A meta-analysis of studies on the association between CAH and spatial ability found that females with CAH perform better on spatial tasks in comparison to control groups^49^. However, the evidence for the role of prenatal testosterone in spatial ability is mixed. Some studies have reported null results when the exposure on prenatal testosterone was measured as a 2D:4D ratio^50,51^. Additionally, one study found no difference in mental rotation performance between CAH females and the control group^45^.

Levels of prenatal testosterone can be measured in amniotic fluid. One study found an association between higher testosterone levels in amniotic fluid and better mental rotation ability for girls at age 7^52^. However, the sample and effect sizes in amniotic fluid studies are small, calling for further investigations to have confidence in the results^53^. Additionally, studies utilizing twin samples have found evidence for a beneficial effect for females of having a male twin. Several studies have explored the Twin Testosterone Transfer Hypothesis^36,54,55^. According to the TTT, having a male co-twin improves females’ spatial ability due to the transmission of prenatal testosterone during gestation. Two studies have supported the TTT hypothesis, showing that females with a male co-twin (Fm) performed better in MRTs than females with a female co-twin (Ff)^54,55^. The first study, based on a single 3D MRT on a sample of 804 twins, showed that females with a male co-twin outperformed females with a female co-twin (*d* = 0.30)^54^. The second study, based on a sample comparing 100 females from fraternal same-sex and 100 females from opposite-sex twin pairs, replicated the results with similar effect size (*d* = 0.38)^55^, giving further support for the TTT hypothesis.

The evidence for TTT from twin samples does not provide a definitive conclusion regarding the etiology of sex differences. Differences in spatial ability between females with a male co-twin and females with a female co-twin could be due to postnatal environmental influences, namely growing up with a brother. For example, some play behaviors have been shown to improve spatial ability^26,56,57^. A review has concluded that playing video games can improve spatial cognition^56^. A study demonstrated that playing ten hours of a video game, requiring spatial skills, significantly improved females’ mental rotation ability (*η*^2^ = 0.39)^26^. Additionally, the positive effects of training on mental rotation have been shown to endure for several months and the improvement was more long-lasting among females^57^. To address the question of the effect of increased participation in spatial activities due to having a brother (not due the transmission of prenatal testosterone), two studies have employed samples of females with non-twin brothers of similar age^55,58^. Both studies found no advantage in mental rotation performance for females with brothers (of similar age) over females with no brothers^55,58^. These results gave indirect support to TTT, suggesting that performance in mental rotation is not influenced by the sex of the sibling via environmental pathways.

The evidence for TTT in spatial ability is currently limited only to mental rotation. The effect of prenatal testosterone on other spatial measures is unclear. A recent study suggested that TTT is not applicable to a range of measures of verbal and non-verbal abilities, in a sample aged between 2 to 16 years^2^. However, the study did not include any spatial ability measures. Additionally, no evidence for TTT was found for mathematical achievement in an adolescent sample^59^. As such, previous research associated prenatal testosterone only with mental rotation performance. The present study fills the gap in the literature by exploring systematically the TTT in relation to fourteen spatial measures (including three tasks involving mental rotation). To achieve this aim, we utilize a large representative sample of twins that provides the statistical power to detect even small effects. We hypothesize that (i) males will perform better than females in all 14 spatial tasks; and that (ii) females with a male co-twin will outperform females with a female co-twin on all tasks.

## Results

### Sex differences in spatial ability

**Figure 1:**
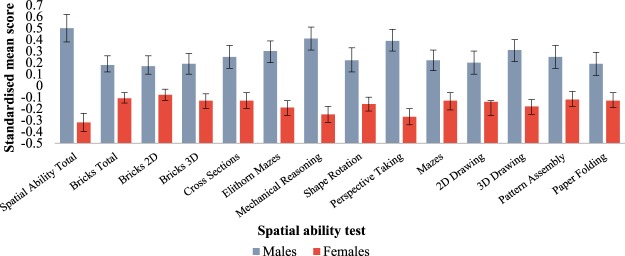
Standardized spatial ability mean scores with 95% confidence intervals, separately for males and females. Note. The means for each task are based on one randomly selected member from each twin pair. All differences were significant at p < 0.01. Effects were considered significant only if they replicated in both halves of the twin sample.

Males outperformed females on all thirteen spatial ability measures (raw scores are available from the authors). To enable meaningful comparisons in sex differences across the measures, the reported values were standardised for the whole sample (males and females combined) with a group mean of 0 and standard deviation of 1. For all measures, males’ average scores were positive and females’ average score were negative, reflecting the overall lower performance of females. The results from the individual tests are plotted in Fig. 1. As shown, the 95% confidence intervals did not overlap between males and females in any of the 14 measures. Overall, the effect sizes were small to moderate (*η*^2^ = 0.02–0.16). The differences for the age-corrected means between males and females maintained their significance after the alpha levels were adjusted to account for the family-wise error rate using the Bonferroni correction (see Supplementary Material S1 for the correlations between all the study measures).

The average mean difference for the overall spatial ability measure, Spatial Ability Total, was significant between males (m = 0.50, sd = 0.92) and females (m = −0.32, sd = 0.90). However, the distributions were largely overlapping. For example, for the Spatial Ability Total, the scores ranged for males between −2.4 and 2.1, and for females between −3.1 and 2.0.

### The twin testosterone transfer hypothesis

**Figure 2:**
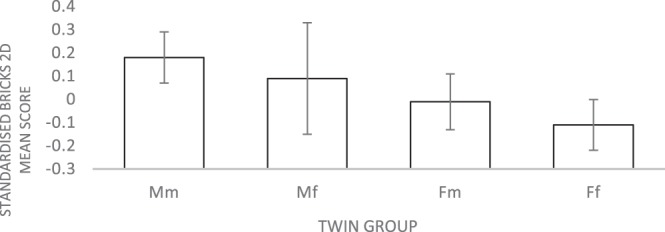
Mean Bricks 2D test scores (with 95% confidence intervals) for males and females from same-sex and opposite-sex twin pairs. Note. Mm = Males with a male co-twin; Mf = Males with a female co-twin; Fm = Females with a male co-twin; Ff = Females with a female co-twin. All the tests were standardized for the whole sample (males and females combined) with a mean of 0. Analyses were then run on these standardized values for males and females separately. For all measures, males’ average scores were positive and females’ average scores were negative.

**Figure 3:**
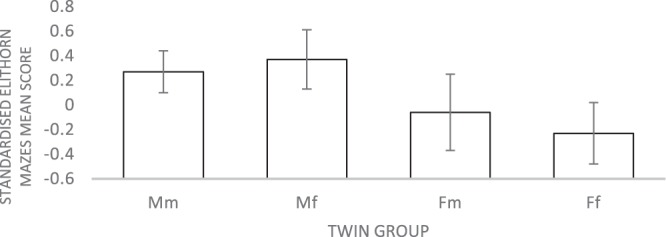
Mean Elithorn Mazes test scores (with 95% confidence intervals) for males and females from same-sex and opposite-sex twin pairs. Note. Mm = Males with a male co-twin; Mf = Males with a female co-twin; Fm = Females with a male co-twin; Ff = Females with a female co-twin. All the tests were standardized for the whole sample (males and females combined) with a mean of 0. Analyses were then run on these standardized values for males and females separately. For all measures, males’ average scores were positive and females’ average scores were negative.

To test the Twin Testosterone Transfer hypothesis on all spatial ability measures, comparisons were conducted between four groups: Mm (males with a male co-twin), Mf (males with a female co-twin), Fm (females with a male co-twin) and Ff (females with a female co-twin). The results showed significant group differences for all measures. To investigate the TTT in detail, post hoc comparisons with Bonferroni correction were carried out between females with a male co-twin (Fm) and females with a female co-twin (Ff). Two of the measures, 2D Bricks and Elithorn Mazes, showed significant average differences between the two female groups. In both measures, Fm group outperformed Ff group. The effect sizes for the differences were very small: Bricks 2D (*η*^2^ < 0.01); Elithorn Mazes (*η*^2^ < 0.02). The means with 95% confidence intervals for the twin groups in 2D Bricks and Elithorn Mazes tasks are plotted in Figs 2 and 3.

**Figure 4:**
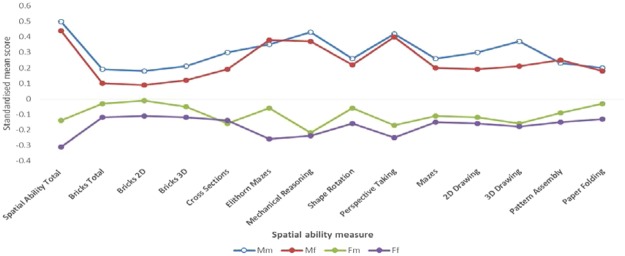
Standardized mean scores for 14 spatial ability scores, separately for four twin groups, based on the sex of the twin and co-twin. Note. Mm = Males with a male co-twin; Mf = Males with a female co-twin; Fm = Females with a male co-twin; Ff = Females with a female co-twin. All the tests were standardized for the whole sample (males and females combined) with a mean of 0. Analyses were then run on these standardized values for males and females separately. For all measures, males’ average scores were positive and females’ average scores were negative.”

The mean scores, standard deviations, F-values and effect sizes for the four twin groups (Mm, Mf, Fm, Ff) on all spatial ability measures are shown in Table 1. To visually assess the group differences, the mean scores are plotted in Fig. 4.

**Table 1:** The age adjusted mean scores, standard deviations, sample sizes, F-values and effect sizes for the four twin groups (Mm, Mf, Fm and Ff) after randomly selecting one twin per pair.

Measure	Mm	Mf	Fm	Ff	F	*η* ^2^
Spatial Ability Total	0.50 (0.92) 342	0.44 (0.92) 167	−0.14 (0.87) 220	−0.31 (0.96) 695	71.41^**^ 1424	0.13
Bricks Total	0.19 (0.71) 699	0.10 (0.72) 360	−0.03 (0.61) 448	−0.12 (0.66) 1410	36.04^**^ 2917	0.04
Bricks 2D	0.18 (0.76) 699	0.09 (0.76) 360	−0.01 (0.70) 448	−0.11 (0.73) 1410	25.64^**^ 2917	0.03
Bricks 3D	0.21 (0.81) 693	0.12 (0.82) 353	−0.05 (0.68) 438	−0.12 (0.76) 1390	31.24^**^ 2874	0.03
Cross Sections	0.30 (1.00) 635	0.19 (1.04) 337	−0.16 (0.96) 446	−0.14 (0.96) 1290	37.89^**^ 2708	0.04
Elithorn Mazes	0.35 (0.93) 556	0.38 (0.89) 296	−0.06 (0.87) 373	−0.26 (1.02) 1077	66.75^**^ 2302	0.08
Mechanical Reasoning	0.43 (1.00) 621	0.37 (0.95) 329	−0.22 (0.93) 425	−0.24 (0.93) 1236	93.53^**^ 2611	0.10
Shape Rotation	0.26 (0.97) 567	0.22 (0.98) 301	−0.06 (0.95) 395	−0.16 (1.00) 1155	29.84^**^ 2418	0.04
Perspective Taking	0.42 (1.11) 572	0.40 (1.15) 299	−0.17 (0.87) 399	−0.25 (0.83) 1165	86.83^**^ 2435	0.10
Mazes	0.26 (1.00) 580	0.20 (0.95) 306	−0.11 (1.01) 393	−0.15 (0.97) 1132	28.16^**^ 2411	0.04
2D Drawing	0.30 (0.90) 627	0.19 (0.95) 336	−0.12 (0.96) 442	−0.16 (1.04) 1278	36.46^**^ 2683	0.04
3D Drawing	0.37 (0.99) 565	0.21 (1.04) 299	−0.16 (0.92) 388	−0.18 (0.96) 1146	48.91^**^ 2398	0.06
Pattern Assembly	0.23 (1.02) 607	0.25 (0.99) 324	−0.09 (0.96) 420	−0.15 (0.97) 1231	28.83^**^ 2582	0.03
Paper Folding	0.20 (1.00) 589	0.18 (1.04) 313	−0.03 (0.98) 420	−0.13 (0.98) 1198	18.75^**^ 2520	0.02

The total sample size for each task is reported under the F-value.

Note. Mm = Males with a male co-twin; Mf = Males with a female co-twin; Fm = Females with a male co-twin; Ff = Females with a female co-twin; F-value represents the variation explained by the mean differences between four twin groups; Eta-squared (*η*^2^) is the value for the effect size; All the tests were standardized for the whole sample (males and females combined) with a mean of 0. Analyses were then run on these standardized values for males and females separately. For all measures, males’ average scores were positive and females’ average scores were negative.

**p < 0.01.

## Discussion

The first aim of the study was to investigate whether previously found sex differences for spatial ability are present across all aspects of spatial ability. As hypothesized, our findings showed that males performed significantly better than females on all examined tasks. Effect sizes ranged from small to moderate, consistent with the previous research which has shown that men outperform women on spatial ability tasks with advantage of up to one standard deviation^7^. Although sex differences in spatial ability were robust, individual differences within sexes explained far more variance in spatial ability than differences between sexes.

The consistently better performance of males over females in all spatial ability measures provides further indirect support for viewing spatial ability as a unitary construct: sex differences in spatial ability maybe maintained by the general spatial ability factor^19,21^. Future studies could explore the role of spatial anxiety in sex differences in spatial ability. A recent study found a small, but significant sex difference: females demonstrated higher spatial anxiety, on average, when facing spatial tasks^60^.

The present study also investigated systematically the degree to which sex differences in spatial ability could be explained by differences in prenatal testosterone. We tested this hypothesis by examining the potential effects of prenatal testosterone transmission between opposite-sex twins. The hypothesis that the influence of prenatal testosterone would be detected in all spatial tasks, was not supported by our data. Only in 2 of the 14 measures, the average scores of Fm twin group were significantly higher in comparison to the Ff group. The effect sizes of the differences were negligible. One of the tasks with a significant difference between the two female groups was 2D Bricks, which is a measure of mental rotation ability. However, there were no significant mean differences between the female groups in the two other mental rotation tasks (3D Bricks and Shape Rotation). Put together, these results are not in line with the previous research, which showed evidence for the superiority of females from opposite-sex twin pairs over females from the same-sex twin pairs in mental rotation^54,55^.

Several factors may account for the differences in results between the current study and previous studies that provided evidence for TTT on mental rotation. Whereas the two previous studies, conducted in Finland^54^ and Germany^55^, utilized convenience samples, the sample of this study is part of a representative UK twin sample. Additionally, the larger number of participants in the present study increased the power of detecting the potential effect. There were also differences in the used measures and data collection methods. The two previous studies only employed a single mental rotation task and data were collected using paper-and-pencil method^54,55^, whereas the current study utilized 14 different computerized spatial measures. Previous research has shown indications that sex differences in spatial ability may vary as a function of the presented stimuli and data collection method. The strength of the sex differences in spatial tasks have been shown to decrease when the used stimuli was presented as real, 3D models instead of 3D images^61^; and when data was collected in virtual environment instead of pencil-and-paper^62^.

Overall, given the strengths of this study (larger, representative sample; 14 spatial measures), the role of prenatal testosterone in spatial ability can be called into question. As indicated by our results, the effect of prenatally transferred testosterone, from male to female fetus, may be too subtle to influence the development of neurocognitive functions associated with spatial ability. Alternatively, it is plausible that the influence of prenatally transmitted testosterone may be only evident for females whose male co-twin produces high levels of testosterone.

Another way to investigate the relationship between the influence of testosterone and cognitive sex differences is to measure gender as a behavioral measure, namely as sex-typed behavior. A recent study has reported the mediating influence of sex roles between sex and spatial ability performance; the results suggested that sex role identity may be more informative in explaining spatial ability than biological sex alone^63^. Since prenatal testosterone levels have been associated with sex role identity^64^, as well as with interest in male-typed activities^52^ it may be beneficial to investigate the role of perceived sex roles in future studies. Perceived sex role identity could be studied not just in relation to testosterone levels, but also in relation to learning and activities that enhance spatial ability (e.g., if sex role identity mediates the relationship between testosterone levels and engagement in spatial activities).

Understanding the causes of sex differences in spatial ability is essential as they may account for today’s underrepresentation of women in STEM professions. The results of this study add to the consistent finding of males’ better performance in spatial ability. However, the role of prenatal testosterone in spatial ability was not supported.

## Methods

### Data

The Twins Early Development Study (TEDS) sample was used in this study. TEDS is a large, longitudinal twin sample that includes more than 13,000 twin pairs born between 1994 and 1996^65^, representative of the population of England and Wales. A subsample of twins, aged 19 to 21, completed a range of spatial ability tests in two separate collection waves. Data from participants with severe medical conditions, or whose mothers had experienced perinatal complications, were excluded from the analyses. After the exclusions, the sample size included 2,928 individuals. Sample sizes varied between the tasks due to different completion rates for different measures. The combined mean age for the participants in the present study was 19.6 years (*SD* = 0.48).

The data were derived from two spatial ability batteries: Bricks and King’s Challenge test batteries^19,21^. The measures in the Bricks test battery were partly based on the classic mental rotation task and designed to investigate mental rotation and spatial visualisation separately, in both 2D and 3D^21^. The 10 King’s Challenge tasks were selected from the pool of 27 spatial measures that resulted from the literature research^19^. The selection was based on the psychometric properties of the measures^19^. Cronbach’s alphas were calculated for all the items of each measure to evaluate their internal consistency/reliability (see Table S3 in the Supplementary Material).

Bricks is a test battery comprising 6 separate tasks (see Supplementary Material S6 for sample stimuli). Some of the tasks are based on the classic mental rotation task^66^. Three of the tasks are 2D versions and three tasks are 3D versions. Two tasks measure visualization (2D and 3D), two tasks measure rotation (2D and 3D) and two tasks assess the combination of visualization and rotation (2D and 3D). Each test includes 12 items, out of which the nine psychometrically best performing items were scored. Due to their short length, the Bricks subtests are not recommended to be used individually^21^. In line with this, three composite Bricks scores, based on the mean performance, were used in the present study: a 2D composite (the three 2D tasks), a 3D composite (the three 3D tasks) and the overall total Bricks score (all six tasks). Reliabilities for the three composite scores ranged from *α* = 0.70–0.84.

The King’s Challenge is a battery of spatial tests, in which the measures are administered within a game-like narrative to encourage participation^19^. It assesses several aspects of spatial ability, not just mental rotation, and is therefore complimentary to the Bricks test battery. King’s Challenge test battery includes the following ten tests: 1) Cross-Sections, 2) Elithorn Mazes, 3) Mechanical Reasoning, 4) Shape Rotation, 5) Perspective-Taking, 6) Mazes, 7) 2D Drawing, 8) 3D Drawing, 9) Paper-Folding, and 10) Pattern Assembly. Demonstration of the gamified test is available from http://teds.ac.uk/research/collaborators-and-data/public-datasets. In the present study, the reliability of two of the tests was lower than recommended *α* = 0.70 (Mechanical Reasoning *α* = 0.54; Mazes *α* = 0.55). However, in the preliminary testing stage^18^ both tests showed good test-retest reliabilities (Mechanical Reasoning *r* = 0.69, *n* = 46, *p* < 0.001; Mazes *r* = 0.74, *n* = 42, *p* < 0.001); and therefore they were included in the test battery. The reliabilities for the remaining tests in the present study were *α* = 0.78–0.92.

To investigate group differences in overall spatial ability, a Spatial Ability Total measure was created. This measure, based on the Principal Component Analysis, was derived from the primary component loadings of each of the spatial ability measures in this study. Spatial Ability Total explained 46% of the total variance among all the study measures. The values for each spatial measure were assigned with the regression method. The Spatial Ability Total is the combination of standardized scores for each participant based on the scores on each measure weighted by the factor loadings. The sample size for Spatial Ability Total was smaller in comparison to the other measures; Spatial Ability Total was calculated only for participants with complete data (who responded to all 13 spatial measures).

### Preliminary analyses

Preliminary analyses showed significant differences in some of the 14 spatial measures between participants from monozygotic and dizygotic same-sex twin pairs (see Supplementary Material S3). However, the effect sizes of the differences were small and did not remain significant after correcting for the family-wise error rate. To increase power, the MZ and DZss twin groups were combined, separately for males and females. To test TTT, further analyses were conducted between four groups based on the sex of the participant and sex of the co-twin: Mm (males with a male co-twin), Mf (males with a female co-twin), Fm (females with a male co-twin) and Ff (females with a female co-twin).

### Data collection

Data collection took place in spring 2015. The data collection was conducted in two waves, separately for Bricks and King’s Challenge batteries. All the tasks were computerized and completed online on the TEDS website by participants after they were sent login details via e-mail. The study was approved by an ethics committee at King’s College London and it was conducted following the approved guidelines. All participants gave their informed consent. The access to the TEDS data is conditional. The complete data access policy can be viewed at https://www.teds.ac.uk/research/collaborators-and-data/teds-data-access-policy.

### Statistical analyses

The data was checked for normality and no data transformations were needed based on the skewness and kurtosis values. Different measures had different number of items, therefore the total scores for each test were standardized to enable comparisons between the tasks.

To control for the effect of age variation in test performances, comparisons were made using one-way ANCOVAs with age (in months) as a covariate. This study used multiple ANCOVAs to examine each spatial measure separately. This statistical technique was chosen over MANCOVA to explore the potential effect of TTT on spatial ability for each aspect of spatial ability, as previous studies suggested some partial independence of the measures. In a previous study, 42% of the variance across the 10 measures was explained by the first principle component^19^. In addition, the use of independent ANCOVAs enables comparisons with previous studies that used single spatial measures^67^.

Analyses to investigate sex differences (Hypothesis 1) were conducted by randomly selecting one individual from each twin pair (the twin group comparisons for the second half of the sample are reported in the Supplementary Material Figure S1 and Table S5). Random selection of one twin per pair created two independent samples, free from inflated inter-individual similarity observed in twins. This approach makes the sample comparable to other (non-twin) samples used in the literature. In addition, this approach allows for replication of the results in another sample (based on the other twin from each pair). If results are significant in one sample, but not in the replication sample – the significance may be a false positive, or the effect size is so small that it cannot be reliably demonstrated. For the comparisons between the twin groups (Hypothesis 2), the whole sample was used – in order to maximize power.

On some of the measures, the variances between the four twin groups (Mm, Mf, Fm, Ff) had significant differences, as shown by Levene’s test. Therefore, all group comparisons were re-run using non-parametric Kruskal-Wallis analysis. For four of the measures (Elithorn Mazes, Perspective Taking, 2D Drawing and Paper Folding), the variances of the four groups were found to be significantly different, and the groups differed in sample sizes. For this reason, we followed up the main analyses with the Kruskal-Wallis test to confirm the results. These further analyses replicated the findings from ANCOVA. Post hoc group comparisons showed that only in Elithorn Mazes there was a small, significant difference between females from same-sex and females from opposite-sex twin pairs.

## Electronic supplementary material

Supplementary information
