# Supplementary material for: Prenatal testosterone does not explain sex differences in spatial ability
Source: Sci Rep. 2018 Sep 12;8:13653. doi: 10.1038/s41598-018-31704-y (PMC6135826; doi:10.1038/s41598-018-31704-y)
Supplement: Supplementary file 1 — Supplementary information [file 41598_2018_31704_MOESM1_ESM.docx]

**Prenatal testosterone does not explain sex differences in spatial ability**

Teemu Toivainen, Giulia Pannini, Kostas A. Papageorgiou, Margherita Malanchini,

Kaili Rimfeld, Nicholas Shakeshaft, & Yulia Kovas

**Supplementary Material**

**Tables**

Table S1. Bivariate correlations between all study variables.

Table S2. Means and 95 % confidence intervals for 14 spatial measures.

Table S3. PCA factor loadings and internal reliabilities (Cronbach’s α) for 13 spatial measures (excluding Spatial Ability Total).

Table S4. Means, standard deviations and t-values comparing MZ and DZss twins, separately for males and females, on 14 spatial ability measures.

Table S5. Examples of Bricks and King’s Challenge test battery measures.

**Table S1**. Bivariate correlations between all study variables.

|  | 1 | 2 | 3 | 4 | 5 | 6 | 7 | 8 | 9 | 10 | 11 | 12 | 13 | 14 |
| --- | --- | --- | --- | --- | --- | --- | --- | --- | --- | --- | --- | --- | --- | --- |
| 1.SA | 1 |  |  |  |  |  |  |  |  |  |  |  |  |  |
| 2.BT | .84* | 1 |  |  |  |  |  |  |  |  |  |  |  |  |
| 3.B2D | .72* | .86* | 1 |  |  |  |  |  |  |  |  |  |  |  |
| 4.B3D | .75* | .90* | .58* | 1 |  |  |  |  |  |  |  |  |  |  |
| 5.CS | .63* | .43* | .38* | .38* | 1 |  |  |  |  |  |  |  |  |  |
| 6.EM | .70* | .36* | .30* | .34* | .23* | 1 |  |  |  |  |  |  |  |  |
| 7.MR | .67* | .49* | .41* | .45* | .45* | .32* | 1 |  |  |  |  |  |  |  |
| 8.SR | .50* | .57* | .50* | .51* | .41* | .32* | .46* | 1 |  |  |  |  |  |  |
| 9.PT | .66* | .39* | .32* | .36* | .34* | .27* | .39* | .41* | 1 |  |  |  |  |  |
| 10.M | .71* | .39* | .32* | .36* | .24* | .28* | .30* | .37* | .29* | 1 |  |  |  |  |
| 11.2DD | .79* | .53* | .46* | .47* | .45* | .36* | .46* | .48* | .37* | .35* | 1 |  |  |  |
| 12.3DD | .70* | .53* | .46* | .48* | .48* | .36* | .50* | .53* | .44* | .43* | .58* | 1 |  |  |
| 13.PA | .58* | .52* | .43* | .49* | .38* | .33* | .41* | .51* | .34* | .38* | .49* | .52* | 1 |  |
| 14.PF | .46* | .54* | .48* | .47* | .47* | .33* | .46* | .53* | .37* | .32* | .50* | .55* | .48* | 1 |

Note. * p< .01; SA = Spatial Ability Total; BT = Bricks total; B2D = Bricks 2D; B3D = Bricks 3D; CS = Cross-Sections; EM = Elithorn Mazes; MR = Mechanical Reasoning; SR = Shape Rotation; PT = Perspective Taking; M = Mazes; 2DD = 2D Drawing; 3DD = 3D Drawing; PF = Paper Folding; PA = Pattern Assembly. The correlations between the tasks are based on one randomly selected member from each twin pair. Random selection of one twin per pair created two similar singleton samples. Effects were considered significant only if they replicated in both halves of the twin sample.

**Table S2**. Means and 95% confidence intervals for the whole sample, separately for males and females, and for the four twin groups.

| **Measure** | **Whole sample** | **All males** | **All females** | **Mean Mm** | **Mean Mf** | **Mean Fm** | **Mean Ff** |
| --- | --- | --- | --- | --- | --- | --- | --- |
| **Spatial Ability Total** | .00  [-.07, .07] | .50  [.44, .68] | -.32  [-.40, -.24] | .50  [.40, .60] | .44  [.29, .58] | -.14  [-.27, -.02] | -.31  [-.38, -.24] |
| **Bricks Total** | -.01  [-.04, .03] | .18  [.12, .24] | -.11  [-.15, -.07] | .19  [.14, .24] | .10  [.03, .17] | -.03  [-.09, .03] | .-.12  [-.15, -.08] |
| **Bricks 2D** | .01  [-.03, .05] | .17  [.11, .24] | -.08  [-.13, -.03] | .18  [.12, .23] | .09  [.01, .25] | -.01  [-.07, .06] | -.11  [-.15, -.07] |
| **Bricks 3D** | -.01  [-.05, .03] | .19  [.12, .26] | -.13  [-.17, -.08] | .21  [.15, .27] | .12  [.04, .20] | -.05  [-.13, .02] | -.12  [-.16, -.08] |
| **Cross Sections** | .01  [-.05, .06] | .25  [.16, .35] | -.13  [-.19, -.07] | .30  [.23, .38] | .19  [.09, .30] | -.16  [-.25, -.07] | -.14  [-.20, -.09] |
| **Elithorn Mazes** | -.01  [-.07, .04] | .30  [.20, .40] | -.19  [-.26, -.12] | .35  [.27, .43] | .38  [.27, .49] | -.06  [-.16, .04] | -.26  [-.32, -.20] |
| **Mechanical Reasoning** | -.02  [-.07, .04] | .41  [.32, .50] | -.25  [-.31, -.19] | .43  [.36, .51] | .37  [.27, .47] | -.22  [-.31, -.13] | -.24  [-.29, -.19] |
| **Shape Rotation** | -.03  [-.08, .03] | .22  [.12, .32] | -.16  [-.22, -.09] | .26  [.18, .34] | .22  [.11, .33] | -.06  [-.16, .03] | -.16  [-.22, -.11] |
| **Perspective Taking** | -.04  [-.10, .01] | .39  [.28, .50] | -.27  [-.33, -.22] | .42  [.35, .50] | .40  [.29, .51] | -.17  [-.26, -.08] | -.25  [-.31, -.20] |
| **Mazes** | -.01  [-.06, .05] | .22  [.13, .32] | -.13  [-.20, -.06] | .26  [.18, .34] | .20  [.09, .31] | -.11  [-.20, -.01] | -.15  [-.21, -.09] |
| **2D Drawing** | -.02  [-.07, .04] | .20  [.11, .29] | -.14  [-.20, -.07] | .30  [.22, .37] | .19  [.09, .30] | -.12  [-.21, -.03] | -.16  [-.21, -.10] |
| **3D Drawing** | -.01  [-.07, .05] | .31  [.21, .41] | -.18  [-.24, -.11] | .37  [.29, .45] | .21  [.10, .32] | -.16  [-.26, -.06] | -.18  [-.24, -.13] |
| **Pattern Assembly** | .01  [-.05, .05] | .24  [.14, .33] | -.12  [-.19, -.06] | .23  [.15, .31] | .25  [.15, .36] | -.09  [-.18, .01] | -.15  [-.21, -.10] |
| **Paper Folding** | -.03  [-.08, .03] | .18  [.08, .28] | -.13  [-.20, -.07] | .20  [.12, .28] | .18  [-.07, .29] | -.03  [-.13, .06] | -.13  [-.19, -.08] |

Note. Mm = males with male co-twin; Mf = males with female co-twin; Fm = females with male co-twin; Ff = females with female co-twin. The means for each task are based on one randomly selected member from each twin pair. Random selection of one twin per pair created two similar singleton samples.

**Table S3**. Primary component loadings from PCA and internal reliabilities (Cronbach’s alphas) for 13 spatial measures

| Measure | Primary component loading | α |
| --- | --- | --- |
| Bricks Total | .84 | .84 |
| Bricks 2D | .72 | .70 |
| Bricks 3D | .75 | .79 |
| Cross Sections | .63 | .78 |
| Elithorn Mazes | .50 | .83 |
| Mechanical Reasoning | .66 | .54 |
| Shape Rotation | .70 | .84 |
| Perspective Taking | .56 | .84 |
| Mazes | .46 | .55 |
| 2D Drawing | .70 | .79 |
| 3D Drawing | .79 | .92 |
| Pattern Assembly | .67 | .79 |
| Paper Folding | .71 | .84 |

Note. The means for each task are based on one randomly selected member from each twin pair. Random selection of one twin per pair created two similar singleton samples.

**Table S4**. Means, standard deviations and t-values comparing MZ and DZss twins, separately for males and females, in 14 spatial ability measures. Significance values are not corrected for the family-wise error rate.

| Measure | Males | | | Females | | |
| --- | --- | --- | --- | --- | --- | --- |
|  | MZ | DZss | t | MZ | DZss | t |
| **Spatial Ability Total** | .44 (1.03) | .58 (.73) | -1.06 | -.34 (.92) | -.36 (.95) | -.28 |
| **Bricks Total** | .21 (.70) | .23 (.71) | -.19 | -.13 (.63) | -.13 (.63) | .01 |
| **Bricks 2D** | .22 (.71) | .20 (.73) | .27 | -.12 (.72) | -.12 (.72) | -.39 |
| **Bricks 3D** | .20 (.82) | .25 (.83) | -.61 | -.13 (.73) | -.15 (.74) | .38 |
| **Cross Sections** | .23 (1.03) | .35 (.93) | -1.07 | -.18 (.94) | -.01 (.88) | -2.40* |
| **Elithorn Mazes** | .24 (1.07) | .31 (1.02) | -.58 | -.26 (1.01) | -.21 (1.00) | -.59 |
| **Mechanical Reasoning** | .36 (1.02) | .47 (.97) | -.96 | -.31 (.95) | -.14 (.90) | -2.24* |
| **Shape Rotation** | .19 (1.00) | .26 (.98) | -.53 | -.16 (.97) | -.16 (1.00) | .45 |
| **Perspective Taking** | .26 (1.04) | .53 (1.11) | -2.10* | -.36 (.75) | -.20 (.80) | -2.42* |
| **Mazes** | .23 (1.00) | .23 (1.06) | -.04 | -.18 (.96) | -.08 (.93) | -1.29 |
| **2D Drawing** | .20 (1.04) | .25 (.81) | -.43 | -.17 (1.02) | -.11 (.98) | -.73 |
| **3D Drawing** | .34 (1.08) | .40 (.88) | -.48 | -.21 (.94) | -.15 (.97) | -.75 |
| **Pattern Assembly** | .24 (1.04) | .27 (1.04) | -.27 | -.11 (.95) | -.12 (.96) | .13 |
| **Paper Folding** | .15 (1.04) | .22 (.91) | -0.54 | -.18 (.99) | -.13 (.96) | -0.59 |

Note. * p< .05; MZ=twins from monozygotic pairs; DZss=twins from dizygotic same-sex pairs. The means for each task are based on one randomly selected member from each twin pair. Random selection of one twin per pair created two similar singleton samples. Effects were considered significant only if they replicated in both halves of the twin sample.

**Table S5**. Examples of the stimuli and short descriptions of the measures.

| Measure | Example | What kind of ability |
| --- | --- | --- |
| 1. Spatial Ability Total | Based on all the measures | Overall spatial ability; based on the co-variance with all 13 measures |
| 2. Bricks Total | Composite of Bricks 2D and Bricks 3D | Mental rotation and visualisation, Bricks 2D and Bricks 3D combined |
| 3. Bricks 2D | 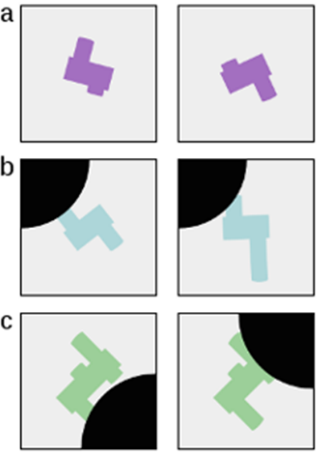 | A measure of 2-dimensional mental rotation and visualisation ability. Participants have to identify the correct 2D stimuli out of four multiple-choice images. |
| 4. Bricks 3D | 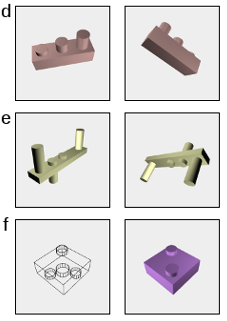 | A measure of 3-dimensional mental rotation and visualisation ability. Participants have to identify the correct 3D stimuli out of four multiple-choice images. |
| 5. Cross  Sections | 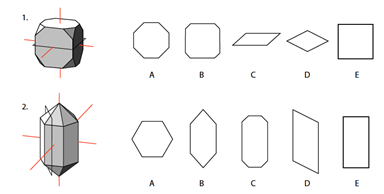 | A measure of spatial reasoning. Participants identify the shape that the cutting plane will produce when cutting through several symmetrical solids (see example figures). The plane can cut the solid vertically, horizontally or obliquely. |
| 6. Elithorn  Mazes | 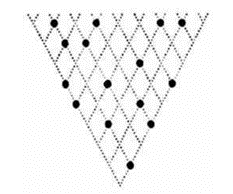 | A measure of spatial planning ability. The aim of the task is to trace the route passing through the largest possible number of black dots on triangular grids.. |
| 7. Mechanical  Reasoning | 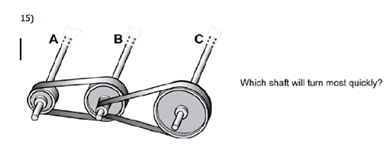 | A measure of ability to understand spatial relations. Examples of questions are: “Which shaft will turn more quickly?” (See example picture) and “If only the right oar of the boat is pulled, in which direction will the boat go?”. |
| 8. Shape  rotation | 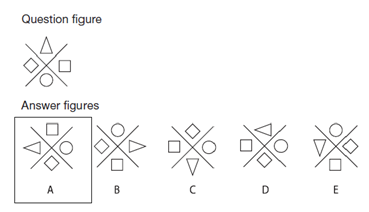 | A measure of mental rotation ability. Participants identify which one of the answer figures (A - E) is the same object as in the question figure, but rotated. |
| 9. Perspective  Taking | 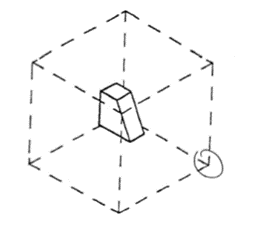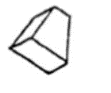 | A measure of spatial orientation. Participants are presented with a transparent cube containing an irregular polygon suspended in the middle of the cube (see example figure). The same polygon is also presented outside the cube from a different viewpoint. Participants indicate on which corner of the cube they would have to stand in order to see the polygon from the new viewpoint (e.g. the bottom right corner in the example figure). |
| 10. Mazes | 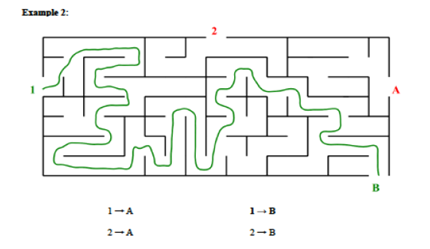 | A measure of navigation ability. Participants are presented with a series of mazes, each with multiple ways in and out, but with only one valid route connecting one of the entrances to one of the exits. Participants look at the map (see example picture on the left) and choose from the options available the valid route between a single entrance and exit. |
| 11. 2D  Drawing | 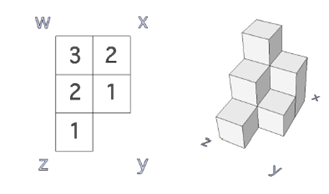 | A measure of spatial manipulation. Participants are presented with the coded plan (see left-hand side of the example picture) and draw the 3D object corresponding to the plan (see right-hand side of the example picture), by clicking on dots arranged in an isometric grid. |
| 12. 3D  Drawing | 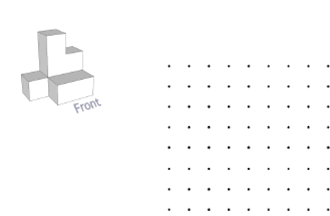 | A measure of spatial manipulation. Participants draw the viewpoint indicated in the picture of the 3D solid as the ‘front’ (see example figure), by clicking on dots arranged in a square grid. The drawing that participants should produce is a 2D viewpoint of the 3D shape. |
| 13. Pattern  Assembly | 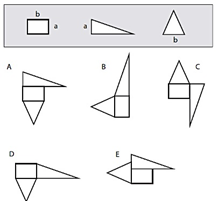 | A measure of spatial reasoning. Participants decide which option (A - E) is made up of the parts presented in the grey box at the top |
| 14. Paper  Folding | 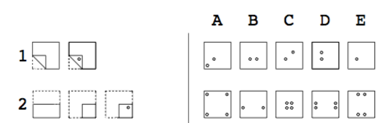 | A measure of spatial reasoning. On the left-hand side of the page participants are shown a sheet of paper folded following several stages. The last image of the sequence includes a dot. This dot represents a hole that is punched through all the thickness of the paper at that point. Participants identify which one of the 5 pictures on the right-hand side shows where the holes will be when the paper is completely unfolded again (by reversing the specific steps shown). |

(Figure and descriptions are adapted from doi:10.1038/srep30545 and doi:10.1073/pnas.1607883114)
